# Supplementary material for: JDroid: Android malware detection using hybrid opcode feature vector
Source: PeerJ Comput Sci. 2025 Jul 25;11:e3051. doi: 10.7717/peerj-cs.3051 (PMC12453807; doi:10.7717/peerj-cs.3051)
Supplement: Supplemental Information 5 [file peerj-cs-11-3051-s005.docx]

**Open source datasets were used in this study. The datasets used are as follows:**

1. MalDroid2020 [44,45]
2. CICInvesAndMal2019 [46]
3. Omer [47]
4. Genome [48]
5. Drebin [49]
